# Increased multiaxial lumbar motion responses during multiple-impulse mechanical force manually assisted spinal manipulation

**DOI:** 10.1186/1746-1340-14-6

**Published:** 2006-04-06

**Authors:** Tony S Keller, Christopher J Colloca, Robert J Moore, Robert Gunzburg, Deed E Harrison

**Affiliations:** 1 Director of Research, Florida Orthopaedic Institute, Tampa, Florida, USA; 2 Master's Candidate, Department Of Kinesiology, Biomechanics Laboratory, Exercise and Sport Science Research Institute, Arizona State University, Tempe, Arizona, USA; Clinic Director, State Of The Art Chiropractic Center, Phoenix, Arizona, USA; 3 Head, The Adelaide Centre For Spinal Research, Institute Of Medical And Veterinary Science, Adelaide, South Australia; 4 Senior Consultant, Department Of Orthopaedic Surgery, Eeuwfeestkliniek Hospital, Antwerpen, Belgium; 5 Vice President, Chiropractic Biophysics Non-profit, Inc., Evanston, Wyoming, USA; Clinic Director, Ruby Mountain Chiropractic Center, Elko, Nevada, USA

## Abstract

**Background:**

Spinal manipulation has been found to create demonstrable segmental and intersegmental spinal motions thought to be biomechanically related to its mechanisms. In the case of impulsive-type instrument device comparisons, significant differences in the force-time characteristics and concomitant motion responses of spinal manipulative instruments have been reported, but studies investigating the response to multiple thrusts (multiple impulse trains) have not been conducted. The purpose of this study was to determine multi-axial segmental and intersegmental motion responses of ovine lumbar vertebrae to single impulse and multiple impulse spinal manipulative thrusts (SMTs).

**Methods:**

Fifteen adolescent Merino sheep were examined. Tri-axial accelerometers were attached to intraosseous pins rigidly fixed to the L1 and L2 lumbar spinous processes under fluoroscopic guidance while the animals were anesthetized. A hand-held electromechanical chiropractic adjusting instrument (Impulse) was used to apply single and repeated force impulses (13 total over a 2.5 second time interval) at three different force settings (low, medium, and high) along the posteroanterior axis of the T12 spinous process. Axial (AX), posteroanterior (PA), and medial-lateral (ML) acceleration responses in adjacent segments (L1, L2) were recorded at a rate of 5000 samples per second. Peak-peak segmental accelerations (L1, L2) and intersegmental acceleration transfer (L1–L2) for each axis and each force setting were computed from the acceleration-time recordings. The initial acceleration response for a single thrust and the maximum acceleration response observed during the 12 multiple impulse trains were compared using a paired observations t-test (POTT, alpha = .05).

**Results:**

Segmental and intersegmental acceleration responses mirrored the peak force magnitude produced by the Impulse Adjusting Instrument. Accelerations were greatest for AX and PA measurement axes. Compared to the initial impulse acceleration response, subsequent multiple SMT impulses were found to produce significantly greater (3% to 25%, *P *< 0.005) AX, PA and ML segmental and intersegmental acceleration responses. Increases in segmental motion responses were greatest for the low force setting (18%–26%), followed by the medium (5%–26%) and high (3%–26%) settings. Adjacent segment (L1) motion responses were maximized following the application of several multiple SMT impulses.

**Conclusion:**

Knowledge of the vertebral motion responses produced by impulse-type, instrument-based adjusting instruments provide biomechanical benchmarks that support the clinical rationale for patient treatment. Our results indicate that impulse-type adjusting instruments that deliver multiple impulse SMTs significantly increase multi-axial spinal motion.

## Background

Spinal manipulation is the most commonly performed therapeutic procedure provided by doctors of chiropractic [1]. Likewise, chiropractic techniques have evolved over the past few decades providing clinicians with new choices in the delivery of particular force-time profiles that are deemed appropriate for a particular patient or condition. In Australia, Canada, and the United States of America mechanical force manually assisted (MFMA) procedures are one of the most popular chiropractic adjusting technique, utilized by approximately 70% of chiropractors [2]. Clinically, single impulse, short duration, MFMA spinal adjustment procedures have been shown to mobilize or oscillate the spine [3-6], elicit neurophysiologic responses [5-10], and enhance acute trunk muscle function [11], However, basic experimental evidence is still lacking that can identify biomechanical mechanisms linked to beneficial therapeutic procedures [12].

Both experimental studies [3,4,13-15] and mathematical models [16,17] indicate that the motion response of the lumbar spine is dependent on the force magnitude, force-time profile and force vector applied. Biomechanical comparisons of hand-held, MFMA-type chiropractic adjusting instruments indicate that the force-time profile (shape, amplitude and duration) significantly affects spinal motion, and suggests that instruments can be tuned to provide optimal force delivery [6,15]. Indeed, a recent animal study [18] demonstrated that oscillatory mechanical forces applied at or near the natural frequency of the lumbar spine are associated with significantly greater displacements (over 2-fold) in comparison to forces that are static or quasi-static. Other animal studies have shown that lumbar spine neuromuscular responses and vertebral displacements are enhanced by increasing force amplitude and pulse duration, while vertebral oscillations (acceleration amplitude and duration) are increased by increasing force amplitude and decreasing pulse duration [6]. We are not aware of any studies, however, that have investigated the biomechanical response of the spine to repeated or multiple impulse MFMA-type mechanical excitation.

The inherent goal of chiropractic adjustments are to induce spinal mobility, therefore research methodology that identifies mechanisms to increase spinal motion is of paramount importance and of great interest to researchers and clinicians. The purpose of this study was to determine the multi-axial segmental and intersegmental motion (acceleration) responses of ovine lumbar vertebral subjected to single and multiple impulse spinal manipulative thrusts (SMTs).

## Methods

### Animal preparation

Fifteen adolescent Merino sheep (mean 47.7 s.d. 4.9 kg) were examined using a research protocol approved by the Animal Ethics Committees and Institutional Review Board of the Institute of Medical and Veterinary Science (Adelaide, South Australia). Sheep were fasted for 24 hours prior to surgery and anesthesia was induced with an intravenous injection of 1 g thiopentone. General anesthesia was maintained after endotracheal intubation by 2.5% halothane and monitored by pulse oximetry and end tidal CO_2 _measurement. Animals were ventilated and the respiration rate was linked to the tidal volume keeping the monitored C0_2 _between 40–60 mmHg.

### Accelerometers

**Figure 1:**
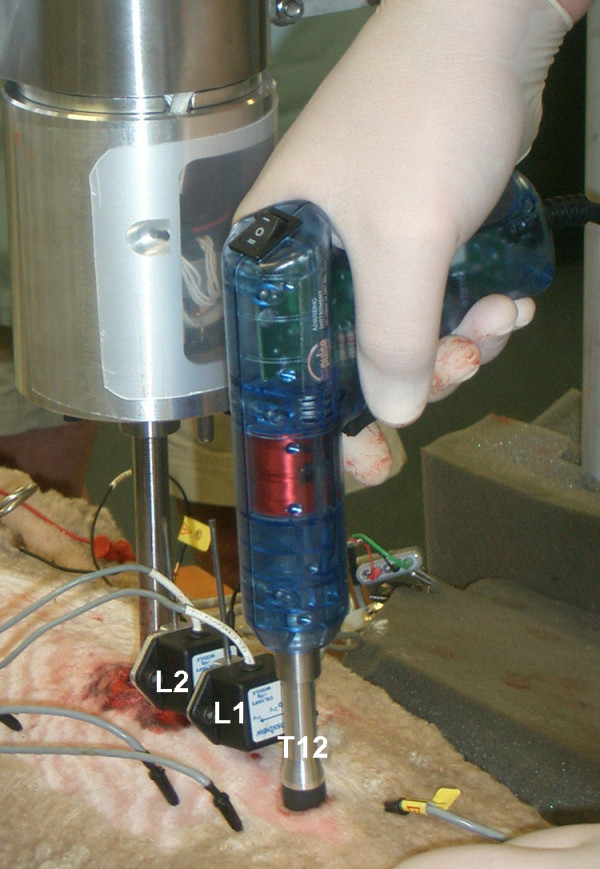
Experimental setup illustrating the Impulse Adjusting Instrument^® ^positioned over the T12 spinous process and the two triaxial accelerometers rigidly attached to stainless steel pins at L1 and L2.

Following anesthesia, the animals were placed in a standardized prone-lying position with the abdomen and thorax supported by a rigid wooden platform and foam padding, respectively, thereby positioning the lumbar spine parallel to the operating table and load frame. Following animal preparation, 10-*g *piezoelectric tri-axial accelerometers (Crossbow Model CXL100HF3, Crossbow Technology, Inc., San Jose, CA) were attached to intraosseous pins that were rigidly fixed to the L1 and L2 lumbar spinous processes under fluoroscopic guidance (Figure 1). The accelerometers are high frequency vibration measurement devices comprised of an advanced piezoelectric material integrated with signal conditioning (charge amp) and current regulation electronics. The sensors feature low noise (300-μ*g *rms), wide bandwidth (0.3 – 10,000 Hz) and low nonlinearity (<1% of full scale) and are precision calibrated by the manufacturer. The *x-*, *y- *and *z*-axes of the accelerometer were oriented with respect to the medial-lateral (ML), posterior-anterior (PA) and cranial-caudal or axial (AX) axes of the vertebrae. The *in situ *natural frequency of the pin and transducer was determined intraoperatively by "tapping" the pins in the ML, PA and AX axes, and was found to be greater than 80 Hertz. This is approximately 20 times greater than the natural frequency of the ovine spine [18], which also exhibits significantly damped motion responses (increased stiffness) for oscillatory PA loads above 15 Hz.

### SMT testing protocol

An Impulse Adjusting Instrument^® ^(Neuromechanical Innovations, LLC, Phoenix, AZ, U.S.A., Impulse) was used to apply posteroanterior (PA) spinal manipulative thrusts to the T12 spinous process of the ovine spine (Figure 1). The T12 spinous process was located by palpation as the first spinous process cephalad to the fluoroscopically verified L1 vertebra containing the accelerometer pin mount. The neoprene end member of the stylus was then placed on the spinous process of T12 and held perpendicularly with a preload of 20 N. Thirteen mechanical excitation impulses were applied over a 2.5 second interval and included a single impulse followed one-half second later by twelve mechanical excitation pulse trains delivered every 160 ms. The Impulse Adjusting Instrument utilizes a microprocessor-controlled electromagnetic coil to produce a haversine-like impulse, approximately 2 ms in duration. Haversine impulse profiles result in a uniform mechanical energy delivery to the test structure over a broad frequency range [6,18], in this case 0 to 200 Hz.

The pulse trains were applied at three different force settings: low (133 N), medium (245 N), and high (380 N). Based upon bench-test experiments, the precision of Impulse device (CoV = standard deviation/mean) was 3.5%, 2.4%, and 1.0% for the low, medium and high force settings, respectively. A doctor of chiropractic with ten years clinical experience administered spinal manipulative thrusts. L1 and L2 vertebral accelerations were recorded at a sampling frequency of 5,000 Hz using a 16 channel, 16-bit MP150 data acquisition system (Biopac Systems, Inc., Goleta, CA, U.S.A.). The sampling period (0.2 ms) was an order of magnitude greater than the Impulse force pulse duration, and the sampling frequency was nearly two orders of magnitude greater than the natural frequency of the pin-accelerometer-bone mount, which ensured that the SMT-induced vertebral oscillations were captured with appropriate signal bandwidth.

### Data analysis and statistics

Acceleration transfer (L1–L2, m/sec^2^, 9.81 m/sec^2 ^= 1-g) between the L1 and L2 vertebrae was estimated by subtracting the L2 accelerometer acceleration-time curve from the L1 acceleration-time curve. The maximum peak-peak acceleration response during the multi-pulse phase (total of 12 pulse trains) was determined and compared to the peak-peak segmental and intersegmental acceleration response obtained during the first impulse. A paired observations t-test was used to determine if the acceleration response during the multi-pulse phase was significantly greater than the initial single impulse (POTT, p < .05 – significant difference). Descriptive statistics (mean, standard deviation S.D.) were also computed, and the changes in motion responses are reported as a percentage of the first thrust.

## Results

**Figure 2:**
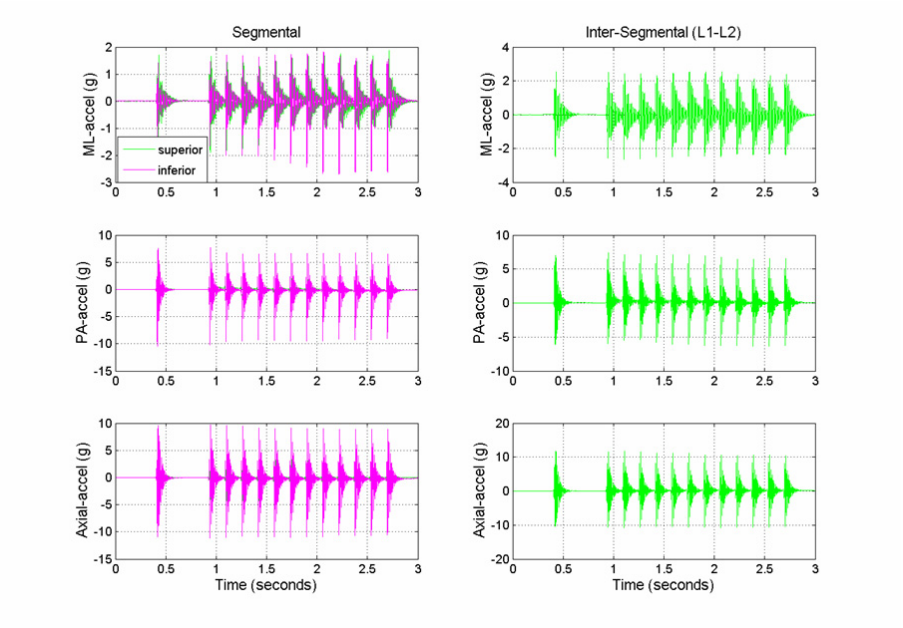
Typical segmental (L1, superior and L2, inferior) and intersegmental (L1–L2) medial-lateral (ML), posterior-anterior (PA), and axial acceleration responses (m/s^2^) during the application of haversine-like mechanical excitation to the ovine spine (high force setting at T12 spinous process, 13 pulse trains).

Typical segmental (L1, L2) and intersegmental (L1–L2) acceleration responses obtained from the multiple impulse adjusting protocol are shown in Figure 2. The short duration (2 ms) mechanical excitation produced by the Impulse Adjusting Instrument^® ^elicited oscillations in the adjacent vertebrae that damped out after approximately 100 to 150 ms. Segmental and intersegmental acceleration responses mirrored the peak force magnitude produced by the Impulse Adjusting Instrument^®^. Accelerations were greatest for AX, followed by PA and ML measurement axes and increased in a linear manner with increasing force magnitude (Table 1). At the highest force setting, the L1 segment ML and PA acceleration responses were 5.6% and 15.4% greater, respectively, in comparison to the L2 segment. The AX accelerations were 17.5% lower at the L1 segment in comparison to the L2 segment (high force setting).

**Table 1:** Initial thrust (impulse thrust 1) mean segmental (L1, L2) and intersegmental (L1–L2) acceleration responses (m/sec^2^). Standard deviations are shown in parentheses.

Impulse Setting	Segment	ML (S.D.)	PA (S.D.)	AX (S.D.)
Low	L1	10.0 (4.0)	36.1 (12.0)	44.6 (11.0)
	L2	8.8 (4.5)	30.7 (10.7)	47.2 (14.8)
	L1–L2	10.1 (3.2)	24.5 (9.3)	39.7 (19.6)
Medium	L1	14.3 (7.1)	71.4 (30.4)	86.7 (31.9)
	L2	14.2 (7.4)	66.2 (21.7)	92.8 (32.9)
	L1–L2	15.3 (6.6)	49.9 (19.5)	81.0 (35.9)
High	L1	27.5 (14.3)	134.4 (46.3)	130.6 (62.8)
	L2	26.1 (14.2)	116.4 (36.1)	158.3 (41.2)
	L1–L2	29.1 (13.1)	107.0 (61.8)	136.8 (64.8)

**Figure 3:**
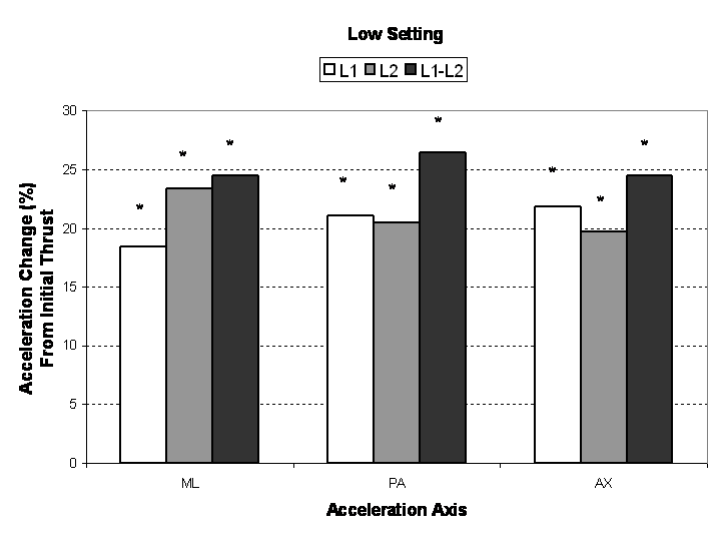
Mean percent change (maximum multi-impulse value compared to first impulse) in *low force*, segmental (L1, L2) and intersegmental (L1–L2) acceleration responses for the medial-lateral (ML), posterior-anterior (PA), and axial (AX) axes. Asterisks (*) indicate significant change from first impulse.

**Figure 4:**
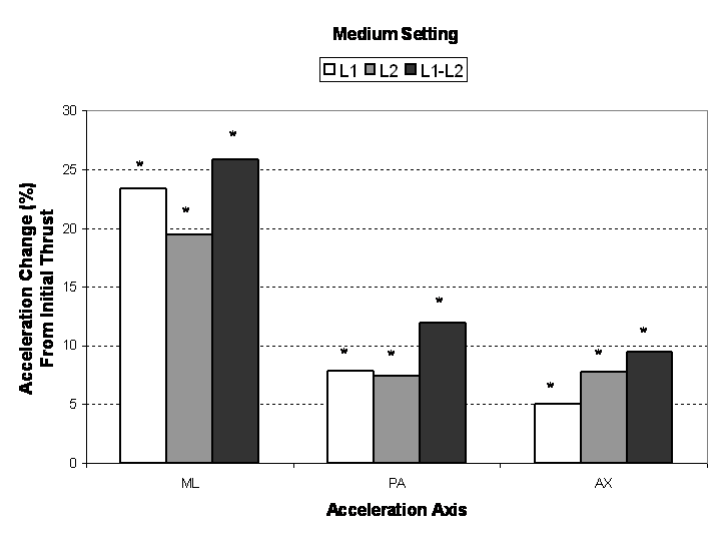
Mean percent change (maximum multi-impulse value compared to first impulse) in *medium force*, segmental (L1, L2) and intersegmental (L1–L2) acceleration responses for the medial-lateral (ML), posterior-anterior (PA), and axial (AX) axes. Asterisks (*) indicate significant change from first impulse.

**Figure 5:**
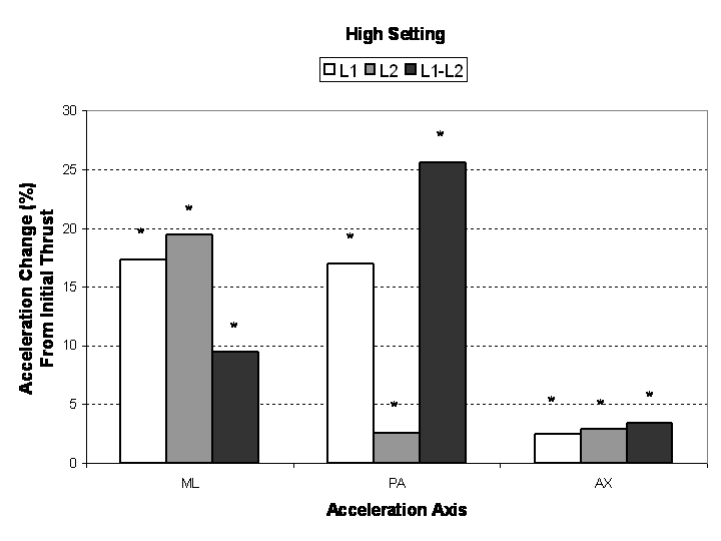
Mean percent change (maximum multi-impulse value compared to first impulse) in *high force*, segmental (L1, L2) and intersegmental (L1–L2) acceleration responses for the medial-lateral (ML), posterior-anterior (PA), and axial (AX) axes. Asterisks (*) indicate significant change from first impulse.

Compared to the initial single impulse acceleration response, subsequent SMT impulses produced significantly greater (3% to 25%, *P *< 0.005) AX, PA and ML segmental and intersegmental acceleration responses (Figures 3, 4, 5). Increases in segmental motion responses (ML, PA, AX) were greatest for the low force setting (18%–26%), followed by the medium (5%–26%) and high (3%–26%) settings. ML, PA and AX motion responses in the L1 segment (adjacent to the applied force) were maximized after the 7^th^, 5^th ^and 3^rd ^SMT impulse (high force setting), respectively. The PA motion response was maximized after the 4^th ^SMT impulse for the low and medium force settings.

## Discussion

Increased segmental and intersegmental acceleration responses were observed when multiple force impulses were applied to the ovine lumbar spine. The increased motion response most likely reflects the dynamic nature of the Impulse Adjusting Instrument^®^, which has a short force-time pulse duration (approximately 2 milliseconds) and causes the ovine spine to oscillate or vibrate for up to 150 ms following the application of the force impulse. The haversine wave shape of the Impulse Adjusting Instrument^® ^creates an efficient mechanical excitation and energy transfer to the spine, which in turn excites a broad range of vibration frequencies (0–200 Hz) in the contacted and adjacent vertebral segments [6]. This frequency range encompassing the resonant frequency (4 Hz) of the ovine spine [18] which, when coupled with the repeated (multiple impulse) mechanical excitation of the spine, amplifies the spinal motion response. Increasing vertebral motions via tuning the frequency and speed of the mechanical inputs during SMT has long been an objective of chiropractic delivery, especially in the development of chiropractic adjusting instruments [16,17,19,20].

A number of studies have quantified the applied forces and concomitant mechanical response of the spine during SMT [9,19-24]. In previous work, we have demonstrated that the stiffness and therefore motion response of different regions of the human [20,25] and animal [18] lumbar spine varied with the mechanical stimulus frequency. Knowledge of the frequency-dependent stiffness characteristics of the spine aids chiropractors in determining the manner in which forces are transmitted to the spine during chiropractic adjustment/spinal manipulation. Such information is important in assessing the biomechanical characteristics of chiropractic treatments, spinal modeling, treatment efficacy, and assessment of risk in the medicolegal arena. To our knowledge, this is the first study to quantify the motion response of the lumbar spine during repeated impulse loading. Our findings indicate that application of multiple short-duration impulses to the spine can increase the magnitude of ensuing vertebral oscillations.

The chiropractic adjusting instrument examined in this study (Impulse Adjusting Instrument^®^) produces a force-time profile with a very short pulse duration (2 ms). Forces that are relatively large in magnitude, but act for a very short time (much less than the natural period of oscillation of the structure), are called *impulsive *[19]. Impulsive forces acting on a mass will result in a sudden change in velocity, but are typically associated with smaller amplitude displacements in comparison to longer duration forces. However, the sudden change in velocity associated with impulsive forces causes the spine to oscillate or vibrate for long periods of time. In the current study we observed that the ovine spine oscillated for a period of time roughly equal to the time interval between impulses (e.g. 160 ms). This corresponds to an impulse loading frequency of 6.25 Hertz, and the application of repeated mechanical excitation resulted in a continuous chain of oscillations in the sheep spine.

The motion response of the spine is closely coupled to the frequency or the time history of the applied force [16]. When external mechanical forces are applied at or near the natural frequency of the spine, greater segmental and intersegmental displacements result (over 2-fold) in comparison to external forces that are static or quasi-static [16]. Thus, it is possible to achieve comparable segmental and intersegmental motion responses for lower applied forces during spinal manipulation, provided that the forces are delivered over time intervals at or near the period corresponding to the natural frequency. Based on the findings of this study, application of repeated mechanical excitation at 6.25 Hertz produces a significantly increased segmental and intersegmental motion response – up to 26% increase in adjacent segment acceleration following the application of several consecutive SMT impulses. Since the oscillations induced in the spine are mostly damped out prior to the onset of the next pulse train, the increased acceleration response is most likely due to mechanical conditioning of the spinal tissues, a desired feature in accomplishing chiropractic adjustment. Noteworthy, axial and medial-lateral accelerations were observed that represent a coupled response to the PA (dorsoventral) forces applied to the ovine spine. We have previously shown that PA thrusts induce coupled motions in both the ML and AX axes [4]. Coupled motions are dependent on a number of factors, including spinal geometry and material properties as well as the force vector applied [16]. As noted in the aforementioned paper, the motion response and coupling are dependent on the intrinsic material properties and geometry, which vary from segment to segment, producing complicated patterns load transmission within the spinal column. Indeed, the decreased axial acceleration response (6–10%) observed for the segment closest to the thrust most likely reflects underlying spinal geometry and material properties. Further research is needed to improve the mechanical excitation characteristics of chiropractic adjustment/spinal manipulation devices and treatment regimes, including force vector, force amplitude, force duration, force-time profile and number of oscillations or impulses applied. We hypothesize that optimization of the mechanical excitation delivered to the spine will enhance neuromechanical and clinical responses in patients.

There are inherent limitations of this study. First and foremost, an animal model was used to study the motion response of the spine. The sheep spine is comprised of structures (ligaments, bone, intervertebral discs) that have qualitatively similar properties as the human spine [26,27], but differ in a number of respects, most notably geometry or morphology. Sheep lumbar vertebrae, and vertebrae of other ungulates (hoofed animals) are more slender and smaller in size compared to human lumbar vertebrae. As a result, the PA stiffness of the ovine lumbar spine is substantially lower (approximately 4-fold) than the human lumbar spine [18]. However, using an animal model we were able to perform invasive measurements of bone movement, which are otherwise difficult to perform in humans [3-5]. Measurement of bone movement using intra-osseous pins equipped with accelerometers [3-5] and other invasive motion measurement devices [28,29] has been previously shown to be a very precise measure of spine segmental motion. Moreover, the short duration (impulsive) mechanical excitation produced very small displacements in the T12 and adjacent vertebrae so the coordinate axes of the vertebrae and accelerometers did not change appreciably. Hence, intersegmental acceleration transfer could be estimated directly from the acceleration-time recordings of the adjacent sensors. However, subtraction of the L1 and L2 time-domain signals to obtain the intersegmental motion response does not take into account the inherent phase differences in the acceleration-time signals. A more comprehensive frequency domain analysis of the acceleration data could be performed [3,16], but this was beyond the scope of this paper.

In addition, testing was performed on anesthetized sheep, so muscle tone was deficient during the tests. The presence of normal or hyper-normal muscle tone may modulate the vibration response of the spine, so we are currently conducting impulsive force measurements while the animals are undergoing muscle stimulation. Finally, vertebral bone acceleration measurements were obtained for vertebrae (L1, L2) adjacent to the point of force application, but we did not quantify the acceleration response of the segment under test (T12) as the accelerometer pin mount and force vector applied precluded contacting the instrumented segment. As a result, the motion amplification response that we observed in adjacent segments following repeated loading may not be representative of the response of the segment under test, which is deemed by most practitioners to be of primary importance. Adjacent segment motion responses, however, are important as it is our belief that the putative effects of MFMA procedures are due to intersegmental motions, which are more similar to intersegmental motions predicted for manual thrusts, as opposed to segmental motions, which are very dissimilar in comparison to manual thrusts [4,5,16,17]. Additional work is needed to quantify both the thrust segment and adjacent segment motion responses to repeated mechanical excitation.

## Conclusion

Our results indicate that repeated multiple-impulse mechanical excitation using an impulsive-type adjusting instrument significantly increases spine motion during the application of multiple impulse SMTs. In principle, mechanical interventions could be tuned to provide specific force delivery for desired biomechanical outcomes including vertebral motion.

## Competing interests

The authors declare that Dr. Colloca is the majority shareholder in Neuromechanical Innovations, LLC, (NMI) the manufacturer of the Impulse Adjusting Instrument^®^. Drs. Colloca and Keller currently hold pending patents specific to the Impulse Adjusting Instrument^®^, whose assignee is NMI. The authors otherwise declare that they have no competing interests.

## Authors' contributions

CC, TK, and DH conceived the study and participated in its design. All authors participated in the collection of data. In the course of data acquisition, RM prepared the animals and oversaw the anesthesia administration and maintenance, RG placed the pins into the spinous processes and affixed accelerometers, and tested their proper working order, CC administered the SMTs, and TK and DH collected and organized the data files. TK performed the statistical analysis, TK and CC drafted the manuscript and DH, RM, and RG edited the manuscript. All authors read and approved the final manuscript.
